# Rediscovering Richard Held: Activity and Passivity in Perceptual Learning

**DOI:** 10.3389/fpsyg.2020.00844

**Published:** 2020-05-19

**Authors:** Fernando Bermejo, Mercedes X. Hüg, Ezequiel A. Di Paolo

**Affiliations:** ^1^Centro de Investigación y Transferencia en Acústica, Universidad Tecnológica Nacional − Facultad Regional Córdoba, CONICET, Córdoba, Argentina; ^2^Facultad de Psicología, Universidad Nacional de Córdoba, Córdoba, Argentina; ^3^Consejo Nacional de Investigaciones Científicas y Tecnológicas, Buenos Aires, Argentina; ^4^Ikerbasque, Basque Foundation for Science, Bilbao, Spain; ^5^IAS Research Center for Life, Mind and Society, University of the Basque Country, San Sebastián, Spain; ^6^Centre for Computational Neuroscience and Robotics, University of Sussex, Brighton, United Kingdom

**Keywords:** Richard M. Held, sensory adaptation, perceptual learning, self-generated movements, activity, passivity, enaction, ecological psychology

## Abstract

Understanding the role of self-generated movements in perceptual learning is central to action-based theories of perception. Pioneering work on sensory adaptation by Richard M. Held during the 1950s and 1960s can still shed light on this question. In a variety of rich experiments Held and his team demonstrated the need for self-generated movements in sensory adaptation and perceptual learning. This body of work received different critical interpretations, was then forgotten for some time, and saw a surge of revived interest within embodied cognitive science. Through a brief review of Held’s work and reactions to it, we seek to contribute to discussions on the role of activity and passivity in perceptual learning. We classify different positions according to whether this role is considered to be contextual (facilitatory, but not necessary), enabling (causally necessary), or constitutive (an inextricable part of the learning process itself). We also offer a critique of the notions of activity and passivity and how they are operationalized in experimental studies. The active-passive distinction is not a binary but involves a series of dimensions and relative degrees that can make it difficult to interpret and replicate experimental results. We introduce three of these dimensions drawing on work on the sense of agency: action initiation, control, and monitoring. These refinements in terms of causal relations and dimensions of activity-passivity should help illuminate open questions concerning the role of activity in perception and perceptual learning and clarify the convergences and differences between enaction and ecological psychology.

## Introduction

Action-based accounts of perception maintain that there are functional and conceptual links between action and perception (e.g., O’Regan and Noë, 2001; Noë, 2004; Pulvermüller and Fadiga, 2010). These perspectives are advocated both by enactivists and ecological psychologists (e.g., Varela et al., 1991; Reed, 1996; Chemero, 2009; Di Paolo et al., 2017) and can serve to highlight the convergences and the differences between these approaches. In the extensive literature on the subject one experiment has become iconic. In a study conducted in 1963, Richard Held and Alan Hein tested the development of visually guided behavior in kittens reared in the dark who were placed in pairs in an illuminated carousel. One of the kittens was “passive” and could not self-locomote. The other was “active” and was free to move by itself while pulling the passive kitten through a transmission mechanism that produced an equivalent visual stimulation. The kittens experienced this condition for 3 h a day for a period of 8 weeks. They were then tested on the visual cliff. Unlike active kittens, passive kittens did not show evidence of differentiating the shallow edge of the cliff from the apparent drop. From this, Held and Hein concluded that self-generated movements are crucial for the development of visual perceptual skills. The experiment is often mentioned in the enactivist and ecological psychology literature (e.g., Gibson, 1969; Varela et al., 1991; Noë, 2004; Heras-Escribano, 2019).

This was not an isolated study. It was part of an extensive research program led by Richard Marx Held (1922–2016) during the 1950s and 1960s. We think that the hypotheses, the innovative experimental designs, and the discussions provoked by these studies are still relevant today. Through a brief review of Held’s work, we seek to contribute to discussions about the role of self-generated movements in perceptual learning. Is the self-generation of movements, or “active movements,” a necessary condition for acquiring new perceptual abilities? Or is it possible to learn to perceive without moving actively, i.e., or through “passive,” externally controlled movements?

Our main conclusion will be that many of the terms in these seemingly clear questions require clarification. In particular, we qualify the terms “active” and “passive” because the discussion will lead us to examine how these notions have been used in experimental psychology and neuroscience. We suggest that what is typically taken as a binary distinction is in fact a spectrum of possibilities with various degrees and different dimensions of activity and passivity. This may be one reason why it is sometimes difficult to replicate experimental results and reach widely accepted conclusions regarding the role of action in perception. We will draw on recent work on the sense of agency to refine the active-passive distinction. We will also offer three distinct meanings for claims concerning the role of activity in perceptual learning.

## Held’s Studies on Sensory Adaptation

Participants in sensory adaptation experiments use interfaces that induce perceptual changes. For example, combinations of special lenses, prisms, and mirrors. The sensory disruption puts in evidence relations between action and perception that otherwise remain hidden. In order to learn to behave and perceive correctly again (in the cases when adaptation occurs), participants must modify their repertoire of sensorimotor schemes. Hermann von Helmholtz and George Stratton performed pioneering studies in visual sensory adaptation in the nineteenth century (Welch, 1974). A few decades later Ewert (1930); Peterson and Peterson (1938) carried out further studies involving long-term exposure and adaptation. The period ranging from the late 1950s to the early 1970s was particularly fruitful with researchers like John G. Taylor, Irving Rock, Hans Wallach making important contributions (Welch, 1974, 1978). According to Welch, the increase in interest may be traced to two sources: the publication by Kohler (1964) of the extensive investigations that he and Theodor Erismann had carried out at the University of Innsbruck, and the work by Held and collaborators. At that time, the team led by Held was setting the pace of the investigation. This period covers his time at Brandeis University (1953–1962) and his first stage at MIT (1962–1971).

During the period in question Held was devoted to the study of adaptation to spatially inverted, reversed, and displaced perception. In most cases he used a technique called *rearrangement*, which consisted in presenting participants with a deliberate distortion of visual or auditory signals through special sensory interfaces. Contemporary studies in sensory adaptation continue to use variations of this method (e.g., Pfordresher and Kulpa, 2011; Bermejo et al., 2020). In typical experiments, Held exposed participants to rearrangement in pretest-posttest designs. To evaluate the effect of self-movement, participants generally underwent an active condition, in which they could move by themselves, and a passive condition, where an experimenter produced in the participant movements equivalent to the active condition. Results, replicated over a series of studies, showed that participants almost invariably compensated for the errors induced by rearrangement only or much more reliably in the active condition (e.g., Held and Hein, 1958; Held and Bossom, 1961; Held and Rekosh, 1963; Held and Mikaelian, 1964).

It is worth taking a brief look at some of these experiments. In one of his first studies, Held (1955) evaluated the effect of experiencing spatially distorted sound cues on sound localization. He used a “pseudophone” that modified the sound streams arriving at the ears causing perceived sounds to be displaced to the left or right. Participants had to orient toward a sound source before and after having practiced with the pseudophone in conditions of self-locomotion. Results showed shifts of auditory localization responses that evidenced a correction of the error induced by the device. Held suggested that adaptation happened because participants were able to associate new interaural patterns with their own movements toward the source. This early formulation of his hypothesis led him to include a passive group in subsequent designs.

In an experiment looking at visual rearrangement, Held and Hein (1958) asked participants to mark with a pencil the apparent location of the corners of a square. They could see their own hands through a prism that produced a lateral visual displacement under different conditions: self-produced hand movement, passive hand movement guided by an experimenter, and no hand movement. The only condition in which the participants were able to compensate for visual displacement was when they could move actively. Held and Bossom (1961) extended the prism rearrangement situation to locomotion. They found that an equivalent correction effect in visual direction-finding occurred when participants performed active self-locomotion. They did not compensate if they were transported by an experimenter in a wheelchair. In a follow-up experiment, Held and Mikaelian (1964) evaluated whether the lack of adaptation in the passive condition could be attributed only to the passivity involved in not being able to initiate movements or in the lack of specificity between movement and contingent stimulation. They replicated Held and Bossom’s (1961) study with the difference that they allowed participants to control the movement of the wheelchair. Adaptation occurred when participants walked by themselves but not when they used the wheelchair. The authors concluded that motor-sensory feedback must correspond to the specific behavior undergoing adaptation, i.e., walking for visual egocentric localization.

These and other studies pointed to similar conclusions^1^, which Held (1965) explained in terms of the active regulation of the plasticity of sensorimotor systems. Situations of rearrangement degrade established patterns of sensorimotor coordination. Under typical conditions, the relation between self-produced movements and stable parts of the environment is univocal and lawful. Each movement has its unique train of sensory stimulations and the perceiver is adapted to this lawful relation. A rearrangement situation alters this relation, confounding participants. A given familiar movement will have unexpected sensory consequences, and expected sensory consequences can only be obtained by the performance of unfamiliar movements. New lawful sensorimotor relations may exist in the rearranged situation but these are not yet obvious to the perceiver. This leads to an increase in performance variability and a decrease in accuracy. As the perceiver explores and practices movements in the rearranged situation, progressive shifts in coordination compensate for the errors induced by the atypical conditions. Gradually, as the new sensorimotor regularities are learned, performance becomes more robust and accurate. This same logic also explains the presence of aftereffects: returning to the original condition after prolonged exposure to the rearranged situation gives rise to errors similar, but in the opposite direction, to those initially induced by rearrangement.

Held consistently finds that self-produced movements are key to adaptation. When movements are passive, the condition of a degraded lawful relation between action and perception does not manifest in the same manner, and consequently adaptation does not occur reliably (Held and Freedman, 1963). “Only an organism that can take account of the output signals to its own musculature is in a position to detect and factor out the rearrangement effects of both moving objects and externally imposed body movement” (Held, 1965, p. 92).

In this context, a key concept is that of re-afferent signals, i.e., stimulation caused by self-produced movements. Exafference, in contrast, is the stimulation that is not induced by the agent’s own movements. Both of these terms are taken from the “efference copy” formulation by von Holst and Mittelstaedt (1950), an influential model explaining the possible mechanisms involved in perceiving the stable attributes of space.

Another key concept is correlation, which is used to describe the relationship between self-produced movements and re-afference. Because in a stable environment there is a unique feedback signal for any particular movement, it is expected that as time passes, the cumulatively experienced efferent and re-afferent signals will show high correlation. The quasi-constant relationships in correlations give rise to the idea of a motor-sensory feedback loop. Sensorimotor coordination of perceptual systems is grounded in the information entailed in these loops (Held and Freedman, 1963).

Held and Freedman (1963) considered these adaptive mechanisms to underlie perceptual learning at any stage of life. At this point, the proposal became a “general theory of the plastic sensorimotor systems” (Held and Freedman, 1963, p. 455) in which the same sensorimotor coordination mechanism is involved in three processes: “(1) the development of normal sensory-motor control in the young, (2) the maintenance of that control once it has developed and (3) the adaptation to changes or apparent changes in the data reported by the senses of sight and hearing” (Held, 1965, p. 84).

Held and colleagues tested this theory by looking at sensorimotor development in young animals. The already mentioned kittens study by Held and Hein (1963) was one in this experimental series. In follow-up studies, Hein et al. (1970) evaluated three hypotheses derived from the carousel experiment. First, they investigated whether the deficit of the passive kittens in the acquisition of visually controlled behavior was due to a facilitating effect of self-locomotion or whether it was impeded by passive transport. To test this, they implemented a similar set-up, with the difference that after being exposed to the passive condition, kittens were allowed to experience the active role for a few hours a day over several days. The results showed that previous exposure to passive transport led to a significant delay in the number of hours needed to acquire a simple visuo-motor capacity (limb extension towards an approaching broad surface) with respect to the group that could always self-locomote.

The authors conducted a second study to determine if passive kittens failed in the visual cliff due to a generalized inhibition of locomotion behavior in response to visual stimuli. In this case, they exposed each eye under a different condition: “periods when visual stimulation of one eye accompanied self-produced movement alternated with periods when visual stimulation was provided to the other eye during passive transport” (Hein et al., 1970, p. 184). As predicted, when kittens could use the eye exposed in the active condition, they were successful both in extension response tests and in the visual cliff. When the same kittens had to use the other eye, they failed in both tasks.

A third experiment extended a study by Hein and Held (1967). When kittens who are prevented from seeing their paws during rearing are carried down toward the edge of a horizontal surface, they show fragmented visual control of their forelimbs (extension but not accurate guidance). Failure to develop guided reaching did not affect the use of the limbs for visually guided locomotion, suggesting that reaching is a separate kind of visuo-motor coordination. In the new experiment, Hein et al. (1970) tested whether visually guided reaching might be acquired independently by each eye. The results showed that when the kittens used the eye that had not received visual input from their own limbs, they failed in visually guided reaching and succeeded when using the other eye. Visually guided reaching did not transfer interocularly.

These follow-up studies support and refine Held’s proposal concerning the enabling role of active movements in perceptual learning, showing that the control of visually guided behavior can be acquired independently for each eye and that an unsystematic correlation between self-movement and visual stimulation produces disruptive effects.

Prior to the kittens experiments, Held made an attempt to adapt his theoretical perspective to von Holst and Mittelstaedt’s (1950) “efference copy” model. Briefly, this model proposes that at the time of motor preparation, copies of the efferent motor information are used to calculate the sensory changes expected as a consequence of its execution. After the effective execution, a real proprioceptive feedback is generated, which will balance the predicted sensory feedback at the level of a so-called “Comparator.” In the case of not finding differences between the two signals, the changes in the afferences would be due to the re-afferences. Otherwise, the changes would be the result of the exafferences. Held (1961) proposed to supplement this model with mechanisms that account for the possibility of adapting to rearrangement situations. To do this, he conceived of an instance before the Comparator stage, which he called Correlation Storage. This module would be responsible for retaining combinations of concurrent efferent and re-afferent signals. The selection of a determinate efferent signal activates a combination that then passes on to the Comparator. In cases of adaptation, correlations of signals must be permanently updated. If the combinations are systematically changed it may happen that the same efferent signal can activate different combinations. This ambiguity will be gradually eliminated as more recent combinations gain more weight.

Evidently this model distances Held’s proposal from perspectives such as ecological psychology and enactivism due to its reliance on internalist explanations. However, Held himself did not make much explicit use of this model. To understand his proposal more fully, it is necessary to take into account that:

(a) In the 1950s and 1960s models such as the “efference copy” were in full swing. Simultaneously, von Holst and Mittelstaedt (1950) and Sperry (1950) had proposed equivalent models and researchers such as Hans-Lukas Teuber, who was head of Held’s department at MIT, were working on these theories. It is likely that this context encouraged Held to reformulate his ideas in terms of comparator models.
(b) The model was only described in his first theoretical work (Held, 1961). In future articles, he did not return to it. Since then his explanations were formulated in terms of self-produced movements, reafference, and the correlation between them, without any mention of instances such as Correlation Storage.
(c) Held clearly rejected internalist interpretations. Immediately after describing the model in the 1961 paper, he remarked that his view of perception does not fit with the idea of the information processor:

“The proposed system neither selects nor filters the incoming signals it receives on the basis of special functional relations or orderings (other than temporal) between the efferent and afferent signals. We can hope that the model forestalls assertions that the nervous system actively seeks a special kind of order which it may store for future reference. Such statements seem to me to beg the issue by assuming an internal “intelligence” to accomplish precisely what requires explanation. (…) If models of the sort proposed convincingly account for adaptive, as well as non-adaptive, psychological processes, we need have recourse neither to a mysterious internal “intelligence” that somewhat knows how to recognize and select useful sensory information nor to the equally mysterious external “intelligence” that manages to reinforce just those responses to sensory stimuli that will prove useful to the organism” (Held, 1961, pp. 31–32).

We may summarize Held’s proposal for explaining sensory adaptation by these claims:

• Self-produced movements are necessary for developing a perceptual ability.
• The stable perception of the environment relies on the consistent coordination of sensorimotor loops.
• Sensory adaptation involves, at first, the disruption of previously established sensorimotor coordination. This is followed by the active gradual reconstruction of new stable sensorimotor patterns.
• The structure of reconstructed sensorimotor loops is constrained by bodily and environmental features and depends on the particular patterns of active practice.
• These principles are not restricted to the visual system, but form part of a general model of perceptual learning.

## Some Reactions to Held’s Proposal

Given the interest raised by Held’s experiments, many contemporary and later studies attempted to replicate their results. Some of them had difficulty doing so. Some found no significant differences in the adaptation achieved between the conditions of passive and active movements (Weinstein et al., 1964; Pick and Hay, 1965; Singer and Day, 1966; Fishkin, 1969; Foley and Maynes, 1969; Baily, 1972; Gyr et al., 1979). There were even studies that showed the possibility of adaptation without movements at all (Howard et al., 1965; Kravitz and Wallach, 1966). The kittens study was particularly difficult to replicate. Walk et al. (1988) wondered if passive kittens paid less attention than active ones to the environment through which they were transported. They repeated the experiment with kittens that either were given something interesting to watch (toy cars racing on a track) or were allowed to move through the environment by lifting their heads to close a microswitch that operated a go-cart. Kittens that paid greater attention to the environment discriminated depth on the visual cliff, whereas those reared with similar light exposure conditions but without the increased attention did not discriminate. The results confirmed the authors’ hypothesis, passive kittens paid less attention than active kittens to the environment and probably this, and not the absence of self-generated movements, enables the learning of spatial skills. It is worth clarifying that kittens were “passive” in the sense used by Held, since they could not walk, but they were “active” in the sense that they paid attention to relevant/interesting events in the environment, being able to move their head or move according to their control. We will return to this point later.

Another point of confrontation with Held’s work concerned rearrangement experiments that involved adapting hand-eye coordination, for example, marking with a pencil the position of a cross that was seen through prisms. Several studies indicated that the perceived discrepancy between the optical image and the postural sensation of the hand was an important cue not taken into account in Held’s explanation of adaptation (Kravitz and Wallach, 1966; Wallach, 1968; Lackner, 1974). According to these studies, the discrepancy is constant and independent whether the hand is moved passively or actively. Therefore, there is no need to re-correlate motor information. The problem is actually one of detecting the constant discrepancy between the true and the seen position of the limb. Adaptation consists in resolving these visual-spatial conflicts. This process involves a great range of factors and subsumes elements like attention, that affect the accuracy of registering the mentioned discrepancies (Lackner, 1981).

Other action-based perspectives at that time, such as incipient work in ecological psychology, also showed a distancing from Held’s proposal. James Gibson’s early empirical work on sensory adaptation was not equivalent to Held’s. It was more focused on what he called phenomenal adaptation. He had developed diverse experiments on adaptation to negative aftereffects (Gibson, 1933, 1937; Gibson and Radner, 1937). These were simple effects showing that during prolonged looking, adaptation can occur in the perceived curvature and tilt of static lines, the curves tending to become straighter and the tilts tending to become either more vertical or horizontal. These perceptual adaptations, according to Gibson, were a sort of phenomenal normalization to the usual conditions of the physical environment. Beyond these experiments, Gibson’s theoretical developments are closely related to “behavioral” adaptations, as he called them. In particular, he did not think that there were important differences in the perceptual information that could be obtained with self-generated movements and passive movements. While he argued that to perceive it is necessary to establish sensorimotor invariants (Gibson, 1979), it seems that the movements activating those invariants could also be passive. The concept of information flow, understood as the pattern of change sensed by the perceiver (Gibson, 1950), for instance, offers the possibility of explaining sensory adaptation processes through passive movements. According to Gibson, in relation to the sensory adaptation experiments:

“Head-movements would be necessary for isolating these new invariants; perhaps voluntary head movements would help in directing attention to them but passive movements should be sufficient. […] In short, according to this formula there is a way of “finding out” about the environment without necessarily behaving in the sense of performing or executing actions.” (Gibson, 1967/1997, §. 9).

Gibson’s position can also be understood by his considerations on proprioception and exteroception, which he did not consider as two fundamentally different and separate channels of information. The exteroceptors, such as the retina, are sensitive both to changes in the direction of objects that move in the environment and to the flow of patterns produced by the movement of the head. Meanwhile, proprioceptors can account for both externally imposed passive movements and self-generated ones. Gibson abandons the notion of independent and purely exteroceptive or proprioceptive fields, and the main problem becomes one of exhaustively defining the entire array of stimulation, irrespective of the particular receptors involved (Cohen, 1981).

“Proprioception considered as the obtaining of information about one’s own action does not necessarily depend on proprioceptors, and exteroception considered as the obtaining of information about extrinsic events does not necessarily depend on exteroceptors.” (Gibson, 1966, p. 34).

Attentive to Gibson’s work on phenomenal adaptation (in curved and tilted lines), Held and colleagues expanded the study of these effects by exploring the possibility they may be enabled by a motor component as well. In two similar studies, they assessed adaptation to tilt (Mikaelian and Held, 1964) and curvature (Held and Rekosh, 1963) under active and passive conditions using specific prisms that modify these properties. Both studies were very ingenious; participants had to estimate the state of lines before and after exploring a scene with a random array of small spots. This has the effect of removing from the visual environment any lines or curves that could provide normalizing visual cues for straightness or vertical/horizontal orientation. In the active condition, participants could self-locomote with the goggles, while in the passive one they were transported by an experimenter on a wheelchair or a cart along the same route. Both studies confirmed Held’s proposal, only self-generated movements under the transformed condition induced by the goggles make participants compensate for the errors due to the prism. On removing the goggles, they experience an aftereffect of the same magnitude, but in the opposite direction as the prismatic distortion. In the passive conditions the after-effect is much smaller. These findings imply that even processes classified as purely visual or phenomenal, such as adaptation due to normalization effects, can also involve motor factors.

Discussions at the time seem to arrive at the consensus that self-generated movements were not essential to achieve sensory adaptation although their presence could facilitate the adaptive process (Welch, 1978). Irving Rock summarized the state of affairs: “[self-generated] movement is important only because it allows for certain kinds of information to be registered, not because movement *per se* is necessary” (Rock, 1966, p. 42). From the 1970s onward Richard Held abandoned the study of sensory adaptation, as he describes in his autobiography (Held, 2008).

## Later Repercussions of Held’s Work

After a period of relative quiet, today Held’s proposal resonates with contemporary action-based theories of perception. The idea has a strong affinity with the enactive approach according to which perception relies on the mastery of lawful sensorimotor regularities, with self-generated movements being an enabling (and possibly constitutive) condition for developing these sensorimotor schemes or contingencies (O’Regan and Noë, 2001; Noë, 2004; Di Paolo et al., 2017). Varela et al. (1991) brought the kittens experiment to the attention of a younger generation: “This beautiful study supports the enactive view that objects are not seen by the visual extraction of features but rather by the visual guidance of action” (pp. 174–175). This view was inspirational for the field of evolutionary robotics during the 1990s and 2000s, where the role of self-generated activity could easily be appreciated in concrete examples (Nolfi and Floreano, 2000; Harvey et al., 2005; Vargas et al., 2014) particularly in models where perceptual information is generated by a robot’s own activity as in the case of a wheeled robot that uses its own angular velocity while circling objects to discriminate their size (Pfeifer and Scheier, 1999). Self-generated activity was explicitly investigated in developmental robotics, as in a replication of the kittens experiment using real mobile robots (Suzuki et al., 2005).

The fit of Held’s ideas with the contemporary ecological perspective is less clear. Mention of Held’s work is cursory or absent from many important books in this tradition (e.g., Lombardo, 1987; Reed, 1996; Heft, 2001; Chemero, 2009; Turvey, 2019), although it enjoyed some recognition in ecological theories of development (see below). While it is still argued that in order to perceive it is necessary to establish sensorimotor invariant rules (Lobo et al., 2018), it is difficult to find similar pronouncements on the explanatory status of self-generated movements.

Mossio and Taraborelli (2008) have discussed these differences between the two schools. Ecological theories conceive of sensorimotor invariants as a specific transformation of information linked to the perceiver’s motion, but their presence and structure do not rely on the voluntary execution of specific motor schemes. By contrast, for the enactive approach motor schemes are intrinsic constituents of perceptual invariants, and schemes are themselves always constituted by bodily and environmental processes. Perception involves the activation of a network of actual and virtual powers and sensitivities, which by definition cannot be entirely passive, and in the case of honing perceptual skills adaptation processes cannot be divorced from activity *of some kind* (Di Paolo et al., 2017). Ecological psychologists focus on the transformation in the ambient array, while enactivists on the changes produced by active sensorimotor schemes. It is a simplification, but it may be helpful to understand this difference to think that for ecological psychologists the paradigmatic case from which perception in general is explained is vision, e.g., appreciating the affordances of a complex scene, while for enactivists it is touch, e.g., perceiving the softness of a sponge by squeezing it.

This crude characterization serves as a first step in differentiating the enactive and ecological positions. Experimental evidence, however, belies this simple picture. For example, parallax and depth perception in cases of ambiguous optic flows are robustly dependent on voluntary movements by the observer (Wexler and van Boxtel, 2005). Wexler (2003) studied the perception of ambiguous optic flows under voluntary (self-produced head movement), involuntary (movement controlled by an experimenter), and mismatch displacement conditions (the participant moves a wheelchair with her hands). These conditions help disentangle motor signals for action initiation (assumed to be available only in voluntary motion) and proprioceptive and vestibular information. The same optic flow information leads to different perceptions in voluntary vs. mismatch and vs. passive conditions. Wexler observes that the difference cannot be due to external flows or to proprioception alone but depends on the motor command. However, “it is not the mere presence of a corollary discharge, but the details of the motor command that are crucial to spatial vision” (p. 344). Wexler concludes that not only can we not disregard self-motion in spatial vision, but “the observer’s active role in initiating and producing that motion is [also] crucial” (p. 344). This evidence, that could be taken to support an enactive interpretation, could also be interpreted ecologically if the sources of integrated information are extended to include motor commands and other somatosensory signals.

Studies like these show that it is difficult to attribute empirical results as supporting either the simplified versions of the enactive or ecological positions. The evidence used often arises from perceptual situations where sensorimotor schemes are already consolidated. One way to force a contrast between the two perspectives is to analyze the development of sensorimotor invariants or contingencies. Trying to answer how sensorimotor invariants achieve a stable structure can shed light on the role of self-generated movements.

To clarify the discussion, we use the terminology introduced by De Jaegher et al. (2010) to describe different kinds of causal relations giving rise to a phenomenon. A *contextual* factor is one whose alteration changes the manifestation of a phenomenon, but not whether it is manifested or not, an *enabling* factor is causally necessary for a phenomenon to occur, and a *constitutive* factor is an inextricable part of what makes the phenomenon what it is. Accordingly, for ecological perspectives self-generated motion has a *contextual* or at most an *enabling* or instrumental role in the specification of invariants, while for the enactive perspective it has an *enabling* or even *constitutive* role in the consolidation of sensorimotor schemes.

From an enactive perspective, perception is constituted by the skillful use of regularities that govern the ongoing coupling between motor and sensory activity, i.e., sensorimotor contingencies (Noë, 2004). Sensorimotor disruptions present the perceiver with radical obstacles and lacunae. Her established sensorimotor skills suddenly cease to make sense. Perceptual adaptation consists in the re-equilibration of sensorimotor schemes guided by engagement in a particular *activity* and the norms it defines, e.g., whether or not the intended object is reached successfully, quickly, efficiently, and so on. While the changes involved are more radical, the processes themselves are, as in Held’s proposal, continuous with those of ongoing equilibration involved in minor adjustments and recalibration (Di Paolo et al., 2014, 2017). A key element of adaptation and perceptual learning is the need for self-generated activity by the agent. New sensorimotor schemes cannot be learned unless the agent engages the world actively and confronts various breakdowns and tries to recover from them, all within the normative context of the activity itself. The degree and speed of adaptation will depend on whether the new normative situation is radically novel or a modification of established habits. This may explain why adaptation is not observed in conditions of mismatched voluntary movement in experiments by Held and Mikaelian (1964) where participants able to walk normally are asked to move themselves in a wheelchair. The time for adapting to a task with a very different normativity (stipulating the appropriate energy, reach, duration, and coordination of movements) can be expected to be significantly longer. Other things being equal, the enactive approach predicts that adaptation will eventually occur even in such cases (and that people with experience controlling wheelchairs will adapt faster).

Sensory adaptation experiments, either following the rearrangement paradigm or in cases of sensory substitution, are frequently cited in support of the enactive account (Varela et al., 1991; Myin and Degenaar, 2014). Situations of rearrangement make apparent the established relation between sensorimotor schemes and perception by distorting it. In order to perceive correctly again this relation must be re-established. The perceiver must modify her sensorimotor schemes. Action and perception are constituted as an *internal* relation between two terms: the perceiver’s repertoire of skills and sensitivities, and the environment. (By internal relation we mean a relation on which the related terms depend and through which they change together, as opposed to an external relation between already defined and fixed entities.) This relation is enacted in concrete form in each particular situation involving current and historical environmental constraints and opportunities, goals, motivations, and so on. Changing either term induces a breakdown in the organization of this relation. This breakdown cannot be recovered exclusively from the agent’s side or from the environment’s side because a new meaningful coherence must be found between two terms in dynamic flux. For this reason, perceptual adaptation is neither the construction of new correlations that internally rearrange environmental data nor the discovery of pregiven invariants in the environment. Rather, it is the result of re-organizing the internal relation between these terms by simultaneously engaging agents and the world. It is eminently a practical, rather than an intellectual, process.

Perceptual learning demands active engagement by the agent with the environment on two related counts: (1) as the process through which new regularities can be explored and equilibrated, and (2) for establishing situated norms that regulate equilibration (otherwise there is no way *for the agent* to know what counts as success or failure and no way to produce new coherent, task-dependent, sensorimotor relations). For each of these reasons, one causal, the other specifying of what to learn, self-generated activity is both *enabling* and *constitutive* of perceptual learning. This activity will in general take the form of combined voluntary overt motor actions such as locomotion, and covert activity such as modulating the focus of attention. It may even take place largely through non-overt activity, as in the case of patients with locked-in syndrome learning to use a brain-computer interface (Kyselo and Di Paolo, 2015).

Ecological psychology studies on recalibration have discussed the role of activity in terms of its relation with environmental flow. For example, Rieser et al. (1995) induced discrepancies between a participant’s walking speed on a treadmill and the rate of optic flow as the treadmill is dragged by a tractor. After these experiences, they showed evidence of perceptual recalibration that cannot be explained by considering motor activity or environmental flow separately. Instead, it is the product of the participant’s sensitivity to the covariance between the two. The authors state that it is difficult to disentangle the precise influence of each variable and that “much work remains to specify the biomechanical information. For example, is efference important?…Is reafference important…?” (p. 496).

Self-generated activity has been more explicitly recognized in ecological approaches to development. Eleanor Gibson seems to have been more sympathetic to the implications of Held’s work than her husband for whom, as we have seen, self-generated movements could be helpful in perception, but not necessary as such. Eleanor Gibson’s approach to perceptual learning and development is indeed compatible with James Gibson’s ideas, but puts more emphasis on the importance of the organism’s active role in exploring the environment (Adolph and Kretch, 2015). Animal and environment are considered as an interactive reciprocal system in which self-produced movement provides dynamic simultaneous information about oneself and environmental events (Gibson and Pick, 2000; Szokolszky et al., 2019). Eleanor Gibson considered locomotion as one of the major organizing behavioral systems in infancy, which allows the learning of many affordances. According to her view, perceptual development implies learning to detect new affordances as action capabilities change due to changes in the body (Gibson, 1992). In a mutual relation that unfolds developmentally, efficient visually controlled locomotion involves perceiving what a given surface affords, and detecting the information that specifies this affordance requires experience in guiding the body. This experience plays a critical role in perceiving the affordance of a surface for locomotion (Gibson and Pick, 2000). Eleanor Gibson was overall more receptive to discussing and accepting the implications of Held’s work (e.g., Gibson, 1969). She mentions the kittens study in support of her own views:

“Self-produced movement, while guiding locomotion visually, emerged as a critical factor in research with kittens by Held and Hein (1963) […] This finding strengthens the notion that guided action combining visual and kinesthetic information from the action systems involved is essential for the kind of affordance that is being learned” (Gibson and Pick, 2000, p. 113).

Along similar lines, Karen Adoph’s studies on infant locomotion led her to the view that a period of self-produced experience is needed to learn to perceive affordances and avoid the visual cliff (Adolph, 2000; Kretch and Adolph, 2013). The learning experience gained with attaining a given posture (e.g., avoiding risky staircases when crawling) is not automatically transferred when a new motor ability is acquired (e.g., walking). It is necessary to learn to perceive the affordances involved in each case, since the perceptual consequences of moving while crawling or walking are very different. To say that self-produced activity is needed, crucial, or essential for perceptual development amounts to assigning it an explanatory role that is not merely *contextual*, but also *enabling*, i.e., without this activity, perceptual learning would not occur.

More recently, Jacobs and Michaels (2007) have proposed a direct learning approach whereby adaptive changes in perception occur without mediating inferential processing. The theory formally describes the information for learning as a vector field covering the space of all the perception-action couplings that can be used to perform an action. Each point in this space corresponds to a specific coupling. Changes during learning are represented as paths. Perceptual learning in this model involves three processes: the education of intention, the education of attention, and calibration. Through the education of intention agents in a situation with multiple alternatives can improve “in choosing which of the possible perceptions and actions they intend to actualize” (p. 326). The education of attention, a term taken from Gibson (1979), is the process of learning to detect the most useful informational variable, even if intention does not change. Calibration consists of changes in the way the informational variable that is operative at a particular moment is used in perception or action. This model is meant to explain a wide range of phenomena in ecologically relevant and informationally rich situations as well as in simpler experimental situations. Although it does not explicitly address the issue of self-generated movements, it makes a clear reference to the active role of the agent. A recent reading of this work suggests that there is an equivalence between the model and the enactive proposal described by Di Paolo et al. (2017). The similarities include, among others, the point that direct learning requires the active role of the perceiver through the perception-action coupling (Higueras-Herbada et al., 2019).

Why do we find different positions within the ecological perspective in relation to Held’s proposal? We believe that this is because theories of perceptual learning (Gibson, 1969; Gibson and Pick, 2000; Jacobs and Michaels, 2007) make use of a concept of agency that is not emphasized in more orthodox ecological positions (beyond the recognition that the determinants of behavior are not exclusively environmental, see Withagen et al., 2012). This concept has emerged more explicitly in recent decades and is part of ongoing discussions within ecological psychology. In a keynote address on the future of psychology published in 1994, Eleanor Gibson referred to agency as one of the hallmarks of human behavior. She uses the term to describe the case when an organism manifests at least some autonomy and control (Gibson, 1994). Agency, according to her, is manifested in human behavior together with three other fundamental hallmarks: prospectivity, retrospectivity, and flexibility. Prospectivity and retrospectivity help define a particular animal’s region of controllable agency. Prospectivity directs action and attention toward the emerging features of situations. Retrospectivity enables agents to coordinate past experiences with present control. Flexibility in action control refers to the interchangeability of means to achieve the ends of action. From these elements Edward Reed (1996) points out that the actions of agents are not the effects of just any previous cause. “Their actions are part of a stream of regulatory activities that are typically self-initiated and modified and regulated by both internal and external factors.” (Reed, 1996, p. 19). Such ideas are consistent with Chemero’s (2009) proposal that we should not think of affordances as dispositional properties. We should understand them in relational terms instead. Chemero believes that perception and action should always be considered in the context of the agent-environment system. To understand the relationship that an agent establishes with her environment, it is not enough to simply focus on the constraints and regularities that may exist, it is also necessary to focus on how the agent is able to selectively be sensitive to or be invited by some affordances and not others (Bruineberg et al., 2019). In making agency an important concept as well as a topic for further research, this ecological strand finds much in common with the enactive approach, for which the idea of agency is central (e.g., Di Paolo, 2005; Barandiaran et al., 2009; Di Paolo et al., 2017).

The discussion today, as it was in the 1960s and thereafter, is fraught with difficulties that arise from the use of apparently straightforward formulations in the context of very complex phenomena. Held proposed that self-generated movements are necessary for achieving sensory adaptation, for perceptual learning and, in general terms, for perceiving in a stable manner. To this we have seen a range of responses that go from the flat denial that self-generated movements (or movement at all) play a role in perceptual learning, to James Gibson’s interpretation that they may facilitate attention but are not really necessary, to Eleanor Gibson and colleagues suggesting they are indeed necessary as part of a mutual developmental influence between action and perception, and to the enactive view, for which an agent’s activity is not only necessary but is itself an inseparable part of the processes of perceptual learning. We have interpreted these different views in terms of contextual, enabling, and constitutive relations. Table 1 summarizes the possible positions on the causal status of self-generated movements in perceptual learning.

**TABLE 1:** Summary of the different responses to Held’s proposal concerning the causal status of self-generated movements (SGM) for sensory adaptation and perceptual learning.

**Explanatory status**	**Interpretation**	**Proponents**
Contextual	SGM may facilitate perceptual learning, but learning can occur without them	J. J. Gibson, Rock, Welch, Lackner
Enabling	SGM are necessary for perceptual learning and development through reciprocal loops between action and perception. Perceptual learning is not possible without them	E. J. Gibson, Adolph, Noë, O’Regan
Constitutive	SGM, or self-generated activity in general, are an integral part of the processes of equilibration, stability, and formation of new schemes that define perceptual learning	Noë, Di Paolo et al.

*The names mentioned as proponents for each case serve only as examples and are based on specific items of literature (not a whole oeuvre). In some cases, (e.g., Alva Noë) the position may be ambiguous between more than one possibility.*

We will not attempt to settle the debate here, in part because we must still critically examine the notions of activity and passivity that have been used above. As with other ideas in these discussions, this distinction is anything but simple.

## Is There Ever a Purely Passive or Purely Active Condition?

Several attempts to replicate Held’s rearrangement studies were unsuccessful. There is an intrinsic difficulty in determining what counts as active and passive conditions in experimental situations. Simple operational definitions can be deceiving. It may be possible to restrict some body movements (e.g., locomotion, movements of the arm) but minor movements (e.g., head or eye movements) are more difficult to control. For instance, in the Held and Hein (1963) experiment all kittens could move their limbs, the difference was that for the passive group there was no correspondence between limb movement and displacement. Active processes that potentially influence perceptual learning can occur in situations of passivity. Indeed, several of Held’s studies show a marked individual variability in the passive condition. Although most of the passive subjects failed to adapt, some did so partially or even fully (e.g., see Held and Hein, 1958; Held and Bossom, 1961; Hein et al., 1970).

Even in cases where participants are completely immobile or are moved by an external force, it is very hard to account for what they are attending to. In the replication of the kittens study by Walk et al. (1988), animals in the passive condition that were watching the toy cars could not locomote but could freely move their head. The authors downplay the role of these movements and attribute spatial learning to the visual scene that captures the kittens’ attention. These movements, however, were very frequent and enabled kittens to discover “a world in depth” (Walk et al., 1988, p. 251). So, even if they could not perform active locomotion, these subtle exploratory actions elicited by the kittens’ interest in the experimental situation could have contributed to learning. It can be hard to determine whether it was one or the other, head and eye movements or visual attention, that gave rise to learning. It may even be a confounded effect between these factors as presumably attention would have faded quickly if the animals could not explore the scene with head and eye movements.

Similar ambiguities arise when defining what counts as an active condition. There can be important differences in the repertoires of actions that participants can perform, from very rich to extremely poor patterns, from attentive and energetic to distracted and lethargic attitudes. As with attention, it is not always easy to ascertain the level of motivation or fatigue with which participants actively perform a task.

An anecdote from an ongoing experiment by two of the authors serves as an example for the point in question. We have performed a study to compare the effect of active and passive exploration on a task where participants, blindfolded and seated, had to reach toward a sound source located in front of them at different distances (similar task as in Hüg et al., 2019). In the active condition, during a training session participants freely explored the arrangement until reaching and touching the sound source. In the passive condition their arm was moved with a sling by the experimenter until the hand made contact with the source. In the posttest the “active” group showed a more precise performance. The “passive” group showed great variability; some participants improved their performance, others did not and others exhibited strange response patterns. At the end of the experiment, passive participants reported very different experiences. For example, one said that she was practically asleep. Another commented that he was extremely attentive to how his arm was being moved. In general, we could not determine these differences from mere observation.

These ambiguities are also manifested in neuroimaging studies. Passive conditions can differ significantly depending on the protocol, motivation, or attention. If passive movements are mechanically administered by a robot, the brain regions that become activated differ from those involved when an experimenter moves the body of the participant. Van de Winckel et al. (2013) suggest that this occurs because the movements performed by the experimenter are never exactly the same, which stimulates in the participant an awareness and sensory monitoring of the moved body. It is not clear if self-generated and passive movements involve the same brain regions. Some studies show that both common and different areas are activated (Sahyoun et al., 2004; Ciccarelli et al., 2005; Van de Winckel et al., 2013). Others do not find any significant difference (Weiller et al., 1996; Guzzetta et al., 2007).

What may look like a reasonable experimental operationalization can fail to capture relevant aspects of a participant’s activity. Activity does not fully stop simply because participants are instructed not to move by themselves. There is, to an extent, always an active element even in the most passive of conditions provided the participant is indeed awake and capable of regulating attention, emotion, effort, inner speech, etc. Participants in typical passive conditions accept an external control source for their movements. But this can involve active elements such as inhibiting a habitual resistance to such external interventions and remaining vigilant that movements do not become too uncomfortable. There is, in contrast, an inherently passive element in every experimental situation, no matter how freely participants may move, in that they accept and comply with the instructions they are given and do not intervene by altering the experimental set-up.

To confound matters further, attributing responsibility for action can be difficult due to social factors, not only in situations of explicit social interaction (De Jaegher et al., 2010), but in general as experimental instructions, clarifications, unintended suggestions, attitudes toward experimenters, social norms, differences in culture and personality, and so on, all form part of a joint participatory construction of sense (De Jaegher and Di Paolo, 2007). Allowing the intervention of another person over the initiation and regulation of our sensorimotor schemes, as in most experimental passive conditions, is a form of interaction that demands an active kind of acceptance and monitoring (see Di Paolo et al., 2018, pp. 148–149). What a participant does or does not do in active or passive conditions is shaped by the social, linguistic, cultural, material, and technical factors at play in the experiment.

Activity and passivity should not in general be understood as forming a binary distinction. This is the case even in apparently well-defined scenarios where the distinction is applied to a restricted domain, such as whether a movement is self-generated or not. With this, we do not intend to imply that the distinction is useless and should be abandoned. Rather, we think it should be refined. We believe there are different degrees and different dimensions of activity and passivity and that articulating these differences has important theoretical and experimental implications.

A first correction that can bring some clarification is to acknowledge that the distinction is in general only a relative one. Given a series of constraints (instructions, set-up, protocol) it may be perfectly valid to describe a condition as active if it allows a significantly higher degree of choice, control, and engagement by the participant than the passive condition, and vice versa (i.e., “more/less active than …”). If constraints remain fixed, the relative difference between the conditions is expected to be maintained. However, since activity and passivity are defined relative to each other, this makes comparison between different experiments risky because of the difficulty of comparing in detail what counts as active or passive in different labs, set-ups, etc.

A more principled approach to refining the active-passive distinction results from considerations concerning the sense of agency, i.e., the aspects of lived experience that continually tell us whether we are the agents of our actions (see e.g., Synofzik et al., 2008; Gallagher, 2012). The sense of agency is illuminating not only because it involves the experienced aspects of the active-passive spectrum but also because the conceptual complexity is similar. The sense of agency is not an either-or aspect of experience, contrary to what we may think by contrasting clear-cut cases such as moving an arm or having somebody else move it for us. It is a sense with many facets not always easy to disentangle. Some aspects of the sense of agency, particularly the feeling of being involved in an action, are pre-reflective and phenomenologically recessive, that is, in normal circumstances primary awareness is with the action not with who is performing it. In cases of breakdown, interruptions, etc., however, we become more presently aware that it was the action that we ourselves have been performing (“something stopped me in my tracks”). Other aspects of the sense of agency, such as the judgment of agency can be reflective, i.e., when we take an introspective stance in the planning of an action or when monitoring its performance. These aspects can take the form of retrospective conceptual attributions (“I did that”) or ongoing deliberate regulations (“Now I must move this cursor just a bit more to the right”).

Similar differences apply to the active-passive distinction. We can expect both pre-reflective and reflective aspects to be in place, as well as differences to do with the prospective/retroactive and ongoing aspects of the action being performed. Buhrmann and Di Paolo (2017) propose a map of these differences (further elaborated in Di Paolo et al., 2017) and connect the phenomenological aspects with microgenetic processes involving the selection, initiation, control, and equilibration of sensorimotor schemes. The same distinctions can be applied to elucidate the active-passive distinction.

One dimension concerns action initiation. This can involve prospective intentional aspects such as an anticipatory awareness of being in a flow of activity and that a particular action needs to be executed next. At the sensorimotor level action initiation correlates with impulses to start an action as well as a sense of urge or preparation. The dimension of action initiation is to be contrasted with ongoing monitoring and control, where the relevant sense is one of progressing toward the achievement of a goal, adapting to deviations or compensating for unforeseen events and obstacles. Imaging studies confirm that different functional brain regions activate during preparation (before active movement), anticipation (prior to passive movement guided by the experimenter), and execution of movement (Sahyoun et al., 2004).

In an experimental situation, it may be relatively easy to control for action initiation in distinguishing between active and passive conditions although some processes, such as preparatory neural motor potentials corresponding to the intention to move, may be active even if the ensuing movement is passive. These processes can make a difference in perception, e.g., preparation to act has been shown to affect visual discrimination (Craighero et al., 1999; Fagioli et al., 2007). It may be less easy to establish a clear-cut difference between activity and passivity in the dimension of monitoring and control. Movement control can be effectively “handed over” to an external agent but this can be an unstable situation precisely because it is unusual and may demand actively trying not to resist the imposed movement, or trying to accompany it, or attempting to predict what the next stage is going to be. In the case of repetitive movements, the participant may fall into a regular pattern where it is unclear who is in control of the movement. Or indeed, in a fully compliant manner, the participant could be doing none of these things.

Controlling experimentally for monitoring is probably not entirely possible (adding distractions or cognitive loads may help if the point is to minimize attention but depending on the hypothesis being tested this may not be desirable). Participants’ monitoring in a passive condition may range from close scrutiny of what is going on to a total lack of attention. Again, if the task is repetitive, participants’ monitoring in the active condition may recede almost entirely as movements become automatic.

These dimensions of the active-passive distinction−initiation, control, monitoring, (Table 2)—and perhaps others too, should be explicitly considered in terms of experimental design and for explicating which aspects of self-generated activity are theoretically most relevant.

**TABLE 2:** Dimensions of the active-passive distinction discussed in the text (there may be more).

**Dimensions of activity-passivity in SGM**	**Description**
Action/movement initiation	Prospective intentions, urge to act, reflective or pre-reflective awareness about what to do next. Preparatory motor potentials, attention to new goals.
Action/movement control	Pre-reflective sense of smooth control or obstacles and breakdowns. Adaptive equilibration via existing or newly learned schemes. Regulation via spinal circuits, etc.
Action/movement monitoring	Attention, whether focused or peripheral, to actions being performed. Ongoing (pre-reflective or reflective) verification of adequacy to intended goals.

*Along each dimension different aspects of an action or movement can make it relatively more or less active. SGM, Self-generated movements.*

## Conclusion

The studies on sensory adaptation carried out by Richard Held and collaborators in the 1950s and 1960s provide us with a rich material for querying the relations between action and perception and, in particular, the role of self-generated activity in perceptual learning. The focus of heated debates at the time, this work is much less discussed today but clearly still very relevant for modern enactive and ecological psychology perspectives, and for clarifying their convergences and differences.

Many of the questions investigated by Held remain unanswered. This is partly due to the connected difficulties in clarifying the meaning of his proposal and in its empirical testing. We have introduced two refinements that throw light on the situation: one for elucidating the kinds of causal relations that may be at play, another for explicating the active-passive distinction.

To say that active, and not passive, participants demonstrate sensory adaptation in cases of rearrangement can mean different things. Self-generated activity may facilitate learning without it being strictly necessary, or it may be required for learning to occur, or it may itself be an inextricable part of the learning process. We have discussed examples of these different interpretations and proposed that they should be, respectively, categorized into contextual, enabling, and constitutive positions (Table 1).

To say that a participant is active, and not passive, can also mean different things. It generally means that they are allowed to move by themselves in contrast to being moved by others. But this difference is relative and dependent on the experimental conditions. Distinct dimensions of activity can be at play in either active or passive conditions. Active movements are externally constrained by social situations, experimental instructions, and set-ups. Self-initiated activity does not necessarily stop when participants allow themselves to be moved. We have appealed to considerations regarding the sense of agency to refine the active-passive distinction and proposed that at least the following three dimensions be differentiated: action initiation, action control, and action monitoring (Table 2). These are strictly dimensions and not binaries in the sense that different aspects and different degrees of intensity can be at play in each.

These considerations can help us understand apparently contradictory empirical evidence and propose more precise hypotheses. Combining the refinements summarized in Tables 1 and 2 yields 9 possible ways of interpreting the claim that active participants adapt better to sensory rearrangement. We do not suggest that this list is exhaustive but it may be enough to help elaborate more precise ways of articulating the convergences and differences both within and between the enactive and the ecological positions.

Clarifying the active-passive distinction goes beyond the study of perception. It has implications, for instance, in areas such as motor rehabilitation. It is well established that active movement improves the recovery of motor function, but the therapeutic role of applying movements passively is still controversial (Lindberg et al., 2004; Zeng et al., 2018; Noble et al., 2019). Some evidence indicates that proprioceptive input caused by passive movements (controlled by a therapist or assisted by a robot) can contribute to improving motor function through the reorganization of the cortical areas involved in sensory integration (Carel et al., 2000). Others, however, state that passive movement is insufficient and active participation by the patient is required (Hogan et al., 2006). Breaking down activity into the dimensions of action initiation, control, and monitoring could help make sense of these differences and knowing which aspects of activity are therapeutically important could lead to improvements in rehabilitation practices.

We conclude by highlighting again the historical and current importance of Richard Held’s rearrangement studies. His experimental designs were original and imaginative, his theoretical interpretations very innovative for the time. Either by affinity or contrast, current action-based theories of perception owe much to Held’s work. In future work, it would be beneficial to examine other theoretical proposals on adaptation and perceptual learning (such as those by Harris, 1965; Rock, 1966; Wallach, 1987), and the role of memory in such processes (e.g., Glenberg, 1997), as well as related work in computational neuroscience in the light of the classifications introduced here. The dialog between enactivists and ecological psychologists, we believe, can only benefit from the common ground that Held’s studies provide and from understanding his ideas more thoroughly.

## Author Contributions

All authors conceived, designed, and wrote the sections of the manuscript and contributed to manuscript revision, read, and approved the submitted version.

## Conflict of Interest

The authors declare that the research was conducted in the absence of any commercial or financial relationships that could be construed as a potential conflict of interest.

## Footnotes

1 According to the SCOPUS database, Held with his team published 34 articles related to this topic between 1955 and 1974.
